# Aqua­(sulfato-κ*O*)bis­[2-(1,3-thia­zol-4-yl-κ*N*)-1*H*-benzimidazole-κ*N* ^3^]iron(II)

**DOI:** 10.1107/S160053681101405X

**Published:** 2011-04-29

**Authors:** Ying Wang, Chang-Fu Zhuang

**Affiliations:** a Southwest Forestry University, Kunming 650224, People’s Republic of China

## Abstract

In the title compound, [Fe(SO_4_)(C_10_H_7_N_3_S)_2_(H_2_O)], the Fe^II^ cation is sixfold coordinated by four N atoms from two 2-(1,3-thia­zol-4-yl)-1*H*-benzimidazole ligands, one water O atom and one O atom of the sulfate dianion within a slightly distorted octa­hedral geometry. The cations and anions are connected by N—H⋯O and O—H⋯O hydrogen bonds into layers in the *ab* plane.

## Related literature

For the spectroscopic properties of similar complexes, see: Devereux *et al.* (2007 ▶). For the importance and applications of coordination polymers, see: Eddaoudi *et al.* (2002 ▶). 
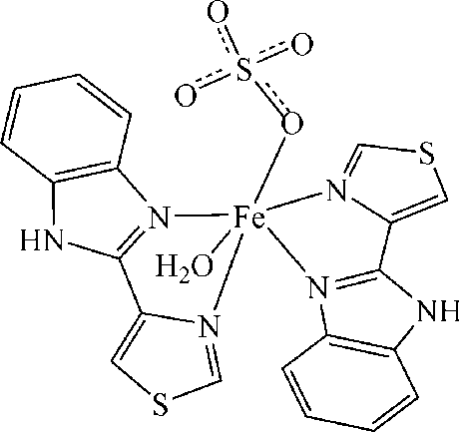


## Experimental

### 

#### Crystal data

[Fe(SO_4_)(C_10_H_7_N_3_S)_2_(H_2_O)]
*M* *_r_* = 572.32
Monoclinic, 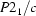

*a* = 12.7401 (6) Å
*b* = 9.7095 (3) Å
*c* = 18.4622 (7) Å
β = 93.518 (2)°
*V* = 2279.47 (15) Å^3^
*Z* = 4
Mo *K*α radiation
μ = 0.98 mm^−1^
*T* = 293 K
0.22 × 0.20 × 0.17 mm

#### Data collection

Bruker SMART CCD diffractometer
Absorption correction: multi-scan (*SADABS*; Bruker, 1997 ▶) *T*_min_ = 0.805, *T*_max_ = 0.846
16163 measured reflections
5632 independent reflections
4071 reflections with *I* > 2σ(*I*)
*R*_int_ = 0.072

#### Refinement

*R*[*F* ^2^ > 2σ(*F* ^2^)] = 0.041
*wR*(*F* ^2^) = 0.114
*S* = 1.03
5632 reflections
316 parameters
H-atom parameters constrained
Δρ_max_ = 1.03 e Å^−3^
Δρ_min_ = −0.39 e Å^−3^

### 

Data collection: *SMART* (Bruker, 1997 ▶); cell refinement: *SAINT* (Bruker, 1997 ▶); data reduction: *SAINT*; program(s) used to solve structure: *SHELXS97* (Sheldrick, 2008 ▶); program(s) used to refine structure: *SHELXL97* (Sheldrick, 2008 ▶); molecular graphics: *SHELXTL* (Sheldrick, 2008 ▶); software used to prepare material for publication: *SHELXTL*.

## Supplementary Material

Crystal structure: contains datablocks global, I. DOI: 10.1107/S160053681101405X/nc2220sup1.cif

Structure factors: contains datablocks I. DOI: 10.1107/S160053681101405X/nc2220Isup2.hkl

Additional supplementary materials:  crystallographic information; 3D view; checkCIF report

## Figures and Tables

**Table 1:** Hydrogen-bond geometry (Å, °)

*D*—H⋯*A*	*D*—H	H⋯*A*	*D*⋯*A*	*D*—H⋯*A*
O1—H1*C*⋯O3^i^	0.75	2.01	2.742 (3)	163
O1—H1*B*⋯O4	0.83	1.90	2.688 (3)	157
N1—H1⋯O4^ii^	0.86	1.92	2.764 (3)	165
N6—H6⋯O5^iii^	0.86	1.90	2.712 (3)	156

Symmetry codes: (i) 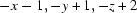; (ii) 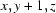; (iii) 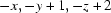.

**Fig. 1.**
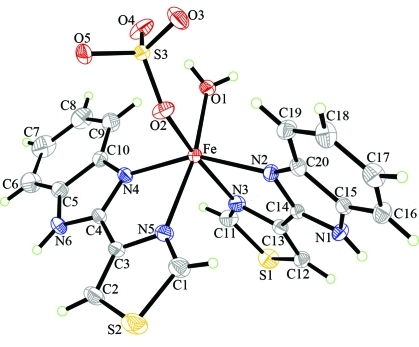
Crystal structure of the title compound with labeling and displacement ellipsoids drawn at the 30% probability level.

**Fig. 2.**
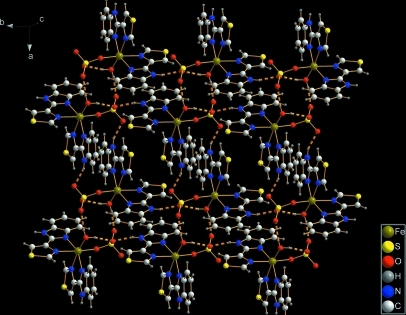
Crystal structure of the title compound with view along the c-axis. Intermolecular hydrogen bonding is shown as dashed lines.

## supplementary crystallographic information

### Comment

Coordination compounds have been extensively studied because of their interesting topologies and potential applications (Eddaoudi *et al.*, 2002). In our own investigations in this field we are interested in compounds based on Thiabendazole, (2-(4'-thiazolyl)-benzimidazole, TBZH) as ligand. Several complexes based on this ligand have been spectrsocopically characterized (Devereux *et al.*, 2007) and only a few compounds have been structurally characterized.

In the crystal structure of the title compound the Fe cation is coordinated by one O atom of one sulfate dianion, one O atom of a coordinated water molecule and four N atoms of two symmetry equivalent TBZH ligands, within slightly distorted octahedra (Fig. 1). The Fe complex cations and the sulfate dianions are connected via O—H···N hydrogen bonding into layers that are located in the a-b-plane (Fig. 2 and Table 1). Additional hydrogen bonds are also found between the water H atoms and the O atoms of the anions as well as the S atoms of the anions.

### Experimental

FeSO_4_7H_2_O (0.279 g, 1 mmol), thiabenzole (0.402 g, 2 mmol), and 16 ml water were mixed with stirring followed by adjusting the pH value to 6.5. Then the mixture was sealed in a 25 ml Teflon-lined stainless steel reactor and heated at 433 K for 96 h to give brown crystals of the title complex after cooling which were dried in air (yield 17% based on Fe).

### Refinement

The H atoms of C— H and N—H were generated geometrically (C—H = 0.93 Å, N—H = 0.86 Å) and refined as riding atoms, with *U*_iso_(H) = 1.2*U*_eq_(C,N). The O—H H atoms were located in difference map and were refined using a riding model with *U*_iso_(H) = 1.5*U*_eq_(O).

### Crystal data

**Table:**

[Fe(SO_4_)(C_10_H_7_N_3_S)_2_(H_2_O)]	*F*(000) = 1168
*M**_r_* = 572.32	*D*_x_ = 1.668 Mg m^−^^3^
Monoclinic, *P*2_1_/*c*	Mo *K*α radiation, λ = 0.71073 Å
*a* = 12.7401 (6) Å	Cell parameters from 102 reflections
*b* = 9.7095 (3) Å	θ = 1.6–28.3°
*c* = 18.4622 (7) Å	µ = 0.98 mm^−^^1^
β = 93.518 (2)°	*T* = 293 K
*V* = 2279.47 (15) Å^3^	Block, brown
*Z* = 4	0.22 × 0.20 × 0.17 mm

### Data collection

**Table:**

Bruker SMART CCD diffractometer	5632 independent reflections
Radiation source: fine-focus sealed tube	4071 reflections with *I* > 2σ(*I*)
graphite	*R*_int_ = 0.072
ω scans	θ_max_ = 28.3°, θ_min_ = 1.6°
Absorption correction: multi-scan (*SADABS*; Bruker, 1997)	*h* = −16→8
*T*_min_ = 0.805, *T*_max_ = 0.846	*k* = −12→12
16163 measured reflections	*l* = −24→24

### Refinement

**Table:**

Refinement on *F*^2^	Primary atom site location: structure-invariant direct methods
Least-squares matrix: full	Secondary atom site location: difference Fourier map
*R*[*F*^2^ > 2σ(*F*^2^)] = 0.041	Hydrogen site location: inferred from neighbouring sites
*wR*(*F*^2^) = 0.114	H-atom parameters constrained
*S* = 1.03	*w* = 1/[σ^2^(*F*_o_^2^) + (0.0542*P*)^2^] where *P* = (*F*_o_^2^ + 2*F*_c_^2^)/3
5632 reflections	(Δ/σ)_max_ = 0.001
316 parameters	Δρ_max_ = 1.03 e Å^−^^3^
0 restraints	Δρ_min_ = −0.39 e Å^−^^3^

### Special details

**Table:**

Geometry. All esds (except the esd in the dihedral angle between two l.s. planes) are estimated using the full covariance matrix. The cell esds are taken into account individually in the estimation of esds in distances, angles and torsion angles; correlations between esds in cell parameters are only used when they are defined by crystal symmetry. An approximate (isotropic) treatment of cell esds is used for estimating esds involving l.s. planes.
Refinement. Refinement of F^2^ against ALL reflections. The weighted R-factor wR and goodness of fit S are based on F^2^, conventional R-factors R are based on F, with F set to zero for negative F^2^. The threshold expression of F^2^ > 2sigma(F^2^) is used only for calculating R-factors(gt) etc. and is not relevant to the choice of reflections for refinement. R-factors based on F^2^ are statistically about twice as large as those based on F, and R- factors based on ALL data will be even larger.

### Fractional atomic coordinates and isotropic or equivalent isotropic displacement parameters (Å^2^)

**Table:**

	*x*	*y*	*z*	*U*_iso_*/*U*_eq_	
Fe	−0.27374 (3)	0.67914 (3)	0.971295 (19)	0.02687 (11)	
S1	−0.26985 (6)	1.03885 (9)	0.79174 (4)	0.0472 (2)	
S2	0.03061 (7)	0.89657 (10)	1.09472 (5)	0.0591 (2)	
S3	−0.31328 (5)	0.40356 (6)	1.07142 (3)	0.02818 (15)	
O1	−0.39228 (13)	0.56116 (18)	0.91389 (10)	0.0349 (4)	
H1C	−0.4489	0.5830	0.9090	0.052*	
H1B	−0.3914	0.4830	0.9325	0.052*	
O2	−0.27394 (15)	0.54526 (17)	1.05838 (10)	0.0397 (4)	
O3	−0.39695 (15)	0.4079 (2)	1.12180 (11)	0.0492 (5)	
O4	−0.35366 (18)	0.34317 (18)	1.00124 (11)	0.0490 (5)	
O5	−0.22550 (14)	0.3195 (2)	1.10114 (12)	0.0458 (5)	
N1	−0.38151 (16)	1.0682 (2)	1.03371 (11)	0.0305 (5)	
H1	−0.3852	1.1537	1.0216	0.037*	
N2	−0.35665 (16)	0.8414 (2)	1.02417 (11)	0.0298 (4)	
N3	−0.28439 (17)	0.8527 (2)	0.88867 (11)	0.0337 (5)	
N4	−0.14511 (15)	0.5944 (2)	0.91289 (11)	0.0298 (4)	
N5	−0.12368 (16)	0.7791 (2)	1.02513 (12)	0.0346 (5)	
N6	0.02727 (17)	0.5786 (2)	0.89849 (12)	0.0375 (5)	
H6	0.0939	0.5906	0.9063	0.045*	
C1	−0.1013 (2)	0.8664 (3)	1.07716 (16)	0.0457 (7)	
H1A	−0.1527	0.9092	1.1029	0.055*	
C2	0.0581 (2)	0.7829 (3)	1.02801 (16)	0.0457 (7)	
H2	0.1253	0.7598	1.0150	0.055*	
C3	−0.03296 (19)	0.7310 (3)	0.99672 (14)	0.0317 (5)	
C4	−0.04923 (18)	0.6333 (3)	0.93656 (13)	0.0302 (5)	
C5	−0.0219 (2)	0.4992 (3)	0.84436 (15)	0.0380 (6)	
C6	0.0172 (3)	0.4221 (3)	0.78838 (19)	0.0600 (9)	
H6A	0.0890	0.4134	0.7831	0.072*	
C7	−0.0562 (3)	0.3589 (4)	0.7409 (2)	0.0678 (11)	
H7	−0.0330	0.3074	0.7026	0.081*	
C8	−0.1629 (3)	0.3702 (3)	0.74911 (17)	0.0589 (9)	
H8	−0.2097	0.3268	0.7159	0.071*	
C9	−0.2019 (2)	0.4442 (3)	0.80516 (16)	0.0464 (7)	
H9A	−0.2739	0.4503	0.8105	0.056*	
C10	−0.1300 (2)	0.5095 (3)	0.85353 (14)	0.0316 (5)	
C11	−0.2584 (2)	0.8733 (3)	0.82185 (14)	0.0387 (6)	
H11	−0.2352	0.8025	0.7929	0.046*	
C12	−0.3125 (2)	1.0873 (3)	0.87508 (14)	0.0343 (6)	
H12	−0.3297	1.1765	0.8883	0.041*	
C13	−0.31676 (18)	0.9747 (3)	0.91818 (13)	0.0294 (5)	
C14	−0.35200 (17)	0.9640 (2)	0.99176 (12)	0.0247 (5)	
C15	−0.40529 (18)	1.0130 (2)	1.10036 (13)	0.0291 (5)	
C16	−0.4362 (2)	1.0730 (3)	1.16334 (14)	0.0370 (6)	
H16	−0.4446	1.1678	1.1675	0.044*	
C17	−0.4537 (2)	0.9849 (3)	1.21975 (14)	0.0413 (7)	
H17	−0.4763	1.0206	1.2629	0.050*	
C18	−0.4386 (2)	0.8451 (3)	1.21381 (15)	0.0445 (7)	
H18	−0.4501	0.7903	1.2539	0.053*	
C19	−0.4071 (2)	0.7810 (3)	1.15094 (14)	0.0375 (6)	
H19	−0.3979	0.6862	1.1479	0.045*	
C20	−0.39029 (19)	0.8695 (3)	1.09280 (13)	0.0300 (5)	

### Atomic displacement parameters (Å^2^)

**Table:**

	*U*^11^	*U*^22^	*U*^33^	*U*^12^	*U*^13^	*U*^23^
Fe	0.02329 (18)	0.02254 (18)	0.0352 (2)	0.00041 (13)	0.00521 (14)	−0.00202 (15)
S1	0.0532 (5)	0.0566 (5)	0.0327 (4)	−0.0033 (4)	0.0094 (3)	0.0103 (3)
S2	0.0460 (5)	0.0673 (6)	0.0625 (5)	−0.0128 (4)	−0.0100 (4)	−0.0237 (5)
S3	0.0238 (3)	0.0258 (3)	0.0347 (3)	−0.0002 (2)	0.0006 (2)	0.0037 (3)
O1	0.0253 (9)	0.0324 (9)	0.0463 (10)	−0.0016 (7)	−0.0038 (8)	0.0043 (8)
O2	0.0505 (12)	0.0280 (9)	0.0398 (10)	−0.0081 (8)	−0.0045 (9)	0.0032 (8)
O3	0.0278 (10)	0.0656 (14)	0.0552 (12)	0.0010 (9)	0.0102 (9)	0.0079 (11)
O4	0.0768 (15)	0.0247 (9)	0.0434 (11)	−0.0056 (9)	−0.0120 (10)	0.0006 (9)
O5	0.0259 (10)	0.0435 (11)	0.0677 (13)	0.0071 (8)	0.0007 (9)	0.0196 (10)
N1	0.0377 (12)	0.0228 (10)	0.0314 (11)	0.0021 (8)	0.0042 (9)	0.0009 (9)
N2	0.0307 (11)	0.0271 (10)	0.0322 (11)	0.0034 (8)	0.0064 (9)	0.0022 (9)
N3	0.0348 (12)	0.0347 (11)	0.0323 (11)	−0.0021 (9)	0.0080 (9)	−0.0036 (9)
N4	0.0237 (10)	0.0308 (11)	0.0348 (11)	0.0000 (8)	0.0003 (8)	−0.0047 (9)
N5	0.0309 (12)	0.0346 (11)	0.0388 (12)	−0.0044 (9)	0.0053 (9)	−0.0041 (10)
N6	0.0231 (11)	0.0427 (12)	0.0477 (13)	0.0013 (9)	0.0099 (9)	−0.0003 (11)
C1	0.0404 (16)	0.0490 (17)	0.0480 (17)	−0.0052 (13)	0.0040 (13)	−0.0140 (14)
C2	0.0282 (14)	0.0527 (17)	0.0553 (17)	−0.0060 (12)	−0.0044 (12)	−0.0095 (15)
C3	0.0250 (12)	0.0321 (13)	0.0375 (13)	−0.0033 (10)	−0.0013 (10)	0.0009 (11)
C4	0.0226 (12)	0.0310 (12)	0.0368 (13)	0.0004 (10)	0.0010 (10)	0.0049 (11)
C5	0.0363 (15)	0.0372 (14)	0.0416 (15)	−0.0009 (12)	0.0118 (12)	0.0016 (12)
C6	0.058 (2)	0.056 (2)	0.069 (2)	0.0000 (17)	0.0328 (18)	−0.0159 (18)
C7	0.092 (3)	0.060 (2)	0.055 (2)	−0.003 (2)	0.034 (2)	−0.0216 (18)
C8	0.078 (3)	0.0530 (19)	0.0449 (18)	−0.0011 (18)	0.0009 (17)	−0.0169 (16)
C9	0.0439 (17)	0.0463 (17)	0.0484 (16)	0.0010 (13)	−0.0025 (13)	−0.0115 (15)
C10	0.0341 (14)	0.0290 (12)	0.0321 (13)	0.0023 (10)	0.0045 (10)	0.0007 (11)
C11	0.0436 (16)	0.0431 (15)	0.0304 (13)	−0.0062 (13)	0.0117 (11)	−0.0060 (12)
C12	0.0349 (14)	0.0326 (13)	0.0356 (13)	0.0005 (11)	0.0039 (11)	0.0018 (11)
C13	0.0262 (12)	0.0314 (13)	0.0307 (12)	−0.0019 (10)	0.0022 (10)	0.0022 (11)
C14	0.0229 (11)	0.0234 (11)	0.0279 (11)	0.0009 (9)	0.0041 (9)	0.0002 (10)
C15	0.0247 (12)	0.0298 (12)	0.0332 (13)	0.0012 (10)	0.0039 (10)	0.0026 (11)
C16	0.0416 (16)	0.0363 (14)	0.0336 (14)	0.0033 (11)	0.0060 (11)	−0.0051 (12)
C17	0.0417 (16)	0.0536 (17)	0.0291 (13)	0.0006 (13)	0.0060 (11)	−0.0020 (13)
C18	0.0463 (17)	0.0546 (18)	0.0330 (14)	0.0092 (14)	0.0061 (12)	0.0028 (14)
C19	0.0425 (15)	0.0320 (13)	0.0388 (14)	0.0010 (11)	0.0102 (12)	0.0069 (12)
C20	0.0275 (12)	0.0345 (13)	0.0282 (12)	0.0019 (10)	0.0043 (10)	−0.0006 (11)

### Geometric parameters (Å, °)

**Table:**

Fe—O2	2.0677 (18)	N6—H6	0.8598
Fe—O1	2.1260 (17)	C1—H1A	0.9300
Fe—N2	2.163 (2)	C2—C3	1.361 (4)
Fe—N4	2.178 (2)	C2—H2	0.9300
Fe—N3	2.272 (2)	C3—C4	1.466 (4)
Fe—N5	2.313 (2)	C5—C6	1.393 (4)
S1—C11	1.704 (3)	C5—C10	1.401 (4)
S1—C12	1.728 (3)	C6—C7	1.384 (5)
S2—C2	1.706 (3)	C6—H6A	0.9300
S2—C1	1.717 (3)	C7—C8	1.382 (5)
S3—O3	1.4577 (19)	C7—H7	0.9300
S3—O5	1.4638 (18)	C8—C9	1.377 (4)
S3—O4	1.485 (2)	C8—H8	0.9300
S3—O2	1.4889 (18)	C9—C10	1.393 (4)
O1—H1C	0.7517	C9—H9A	0.9300
O1—H1B	0.8330	C11—H11	0.9300
N1—C14	1.342 (3)	C12—C13	1.355 (3)
N1—C15	1.392 (3)	C12—H12	0.9300
N1—H1	0.8604	C13—C14	1.460 (3)
N2—C14	1.335 (3)	C15—C16	1.380 (3)
N2—C20	1.389 (3)	C15—C20	1.414 (3)
N3—C11	1.312 (3)	C16—C17	1.377 (4)
N3—C13	1.377 (3)	C16—H16	0.9300
N4—C4	1.327 (3)	C17—C18	1.376 (4)
N4—C10	1.394 (3)	C17—H17	0.9300
N5—C1	1.299 (3)	C18—C19	1.398 (4)
N5—C3	1.380 (3)	C18—H18	0.9300
N6—C4	1.345 (3)	C19—C20	1.402 (3)
N6—C5	1.382 (4)	C19—H19	0.9300
			
O2—Fe—O1	90.80 (7)	N5—C3—C4	115.1 (2)
O2—Fe—N2	94.77 (7)	N4—C4—N6	113.4 (2)
O1—Fe—N2	105.42 (7)	N4—C4—C3	121.2 (2)
O2—Fe—N4	100.63 (8)	N6—C4—C3	125.3 (2)
O1—Fe—N4	94.74 (7)	N6—C5—C6	132.1 (3)
N2—Fe—N4	154.42 (8)	N6—C5—C10	106.1 (2)
O2—Fe—N3	170.30 (7)	C6—C5—C10	121.8 (3)
O1—Fe—N3	93.08 (7)	C7—C6—C5	116.7 (3)
N2—Fe—N3	75.63 (7)	C7—C6—H6A	121.7
N4—Fe—N3	87.91 (8)	C5—C6—H6A	121.7
O2—Fe—N5	88.30 (8)	C8—C7—C6	121.7 (3)
O1—Fe—N5	169.52 (7)	C8—C7—H7	119.1
N2—Fe—N5	85.06 (8)	C6—C7—H7	119.1
N4—Fe—N5	75.18 (7)	C9—C8—C7	121.8 (3)
N3—Fe—N5	89.47 (8)	C9—C8—H8	119.1
C11—S1—C12	89.51 (12)	C7—C8—H8	119.1
C2—S2—C1	89.73 (14)	C8—C9—C10	117.7 (3)
O3—S3—O5	110.41 (12)	C8—C9—H9A	121.1
O3—S3—O4	109.90 (13)	C10—C9—H9A	121.1
O5—S3—O4	108.83 (12)	C9—C10—N4	131.0 (2)
O3—S3—O2	110.05 (12)	C9—C10—C5	120.2 (3)
O5—S3—O2	108.60 (11)	N4—C10—C5	108.8 (2)
O4—S3—O2	109.01 (11)	N3—C11—S1	115.4 (2)
Fe—O1—H1C	123.7	N3—C11—H11	122.3
Fe—O1—H1B	107.1	S1—C11—H11	122.3
H1C—O1—H1B	107.1	C13—C12—S1	109.3 (2)
S3—O2—Fe	136.32 (11)	C13—C12—H12	125.4
C14—N1—C15	107.7 (2)	S1—C12—H12	125.4
C14—N1—H1	126.1	C12—C13—N3	115.9 (2)
C15—N1—H1	126.2	C12—C13—C14	129.0 (2)
C14—N2—C20	105.0 (2)	N3—C13—C14	115.0 (2)
C14—N2—Fe	114.26 (15)	N2—C14—N1	113.1 (2)
C20—N2—Fe	137.82 (16)	N2—C14—C13	120.3 (2)
C11—N3—C13	109.9 (2)	N1—C14—C13	126.6 (2)
C11—N3—Fe	137.40 (19)	C16—C15—N1	132.1 (2)
C13—N3—Fe	112.30 (15)	C16—C15—C20	123.2 (2)
C4—N4—C10	105.0 (2)	N1—C15—C20	104.7 (2)
C4—N4—Fe	115.79 (16)	C17—C16—C15	116.3 (2)
C10—N4—Fe	139.21 (16)	C17—C16—H16	121.9
C1—N5—C3	110.5 (2)	C15—C16—H16	121.9
C1—N5—Fe	136.83 (19)	C18—C17—C16	121.5 (3)
C3—N5—Fe	112.58 (16)	C18—C17—H17	119.2
C4—N6—C5	106.7 (2)	C16—C17—H17	119.2
C4—N6—H6	126.7	C17—C18—C19	123.7 (3)
C5—N6—H6	126.6	C17—C18—H18	118.2
N5—C1—S2	114.8 (2)	C19—C18—H18	118.2
N5—C1—H1A	122.6	C18—C19—C20	115.3 (2)
S2—C1—H1A	122.6	C18—C19—H19	122.3
C3—C2—S2	109.8 (2)	C20—C19—H19	122.3
C3—C2—H2	125.1	N2—C20—C19	130.4 (2)
S2—C2—H2	125.1	N2—C20—C15	109.5 (2)
C2—C3—N5	115.2 (2)	C19—C20—C15	120.0 (2)
C2—C3—C4	129.7 (2)		
			
O3—S3—O2—Fe	−114.18 (18)	Fe—N4—C4—C3	−2.1 (3)
O5—S3—O2—Fe	124.86 (18)	C5—N6—C4—N4	1.2 (3)
O4—S3—O2—Fe	6.4 (2)	C5—N6—C4—C3	−176.3 (2)
O1—Fe—O2—S3	16.14 (18)	C2—C3—C4—N4	−179.7 (3)
N2—Fe—O2—S3	121.68 (18)	N5—C3—C4—N4	−0.8 (3)
N4—Fe—O2—S3	−78.84 (18)	C2—C3—C4—N6	−2.4 (4)
N3—Fe—O2—S3	129.8 (4)	N5—C3—C4—N6	176.6 (2)
N5—Fe—O2—S3	−153.42 (18)	C4—N6—C5—C6	178.3 (3)
O2—Fe—N2—C14	164.86 (17)	C4—N6—C5—C10	−0.6 (3)
O1—Fe—N2—C14	−102.98 (17)	N6—C5—C6—C7	−177.1 (3)
N4—Fe—N2—C14	37.8 (3)	C10—C5—C6—C7	1.7 (5)
N3—Fe—N2—C14	−13.73 (16)	C5—C6—C7—C8	−0.7 (5)
N5—Fe—N2—C14	76.99 (17)	C6—C7—C8—C9	−0.5 (6)
O2—Fe—N2—C20	8.0 (3)	C7—C8—C9—C10	0.8 (5)
O1—Fe—N2—C20	100.1 (2)	C8—C9—C10—N4	177.3 (3)
N4—Fe—N2—C20	−119.1 (3)	C8—C9—C10—C5	0.1 (4)
N3—Fe—N2—C20	−170.6 (3)	C4—N4—C10—C9	−176.6 (3)
N5—Fe—N2—C20	−79.9 (2)	Fe—N4—C10—C9	1.3 (4)
O2—Fe—N3—C11	174.9 (4)	C4—N4—C10—C5	0.8 (3)
O1—Fe—N3—C11	−71.6 (3)	Fe—N4—C10—C5	178.70 (19)
N2—Fe—N3—C11	−176.7 (3)	N6—C5—C10—C9	177.6 (2)
N4—Fe—N3—C11	23.0 (3)	C6—C5—C10—C9	−1.4 (4)
N5—Fe—N3—C11	98.2 (3)	N6—C5—C10—N4	−0.1 (3)
O2—Fe—N3—C13	3.5 (6)	C6—C5—C10—N4	−179.1 (3)
O1—Fe—N3—C13	117.00 (17)	C13—N3—C11—S1	0.7 (3)
N2—Fe—N3—C13	11.86 (16)	Fe—N3—C11—S1	−170.82 (15)
N4—Fe—N3—C13	−148.36 (17)	C12—S1—C11—N3	0.3 (2)
N5—Fe—N3—C13	−73.18 (17)	C11—S1—C12—C13	−1.3 (2)
O2—Fe—N4—C4	−82.69 (18)	S1—C12—C13—N3	2.1 (3)
O1—Fe—N4—C4	−174.40 (17)	S1—C12—C13—C14	−177.3 (2)
N2—Fe—N4—C4	43.3 (3)	C11—N3—C13—C12	−1.8 (3)
N3—Fe—N4—C4	92.68 (18)	Fe—N3—C13—C12	171.99 (18)
N5—Fe—N4—C4	2.67 (17)	C11—N3—C13—C14	177.6 (2)
O2—Fe—N4—C10	99.6 (2)	Fe—N3—C13—C14	−8.6 (3)
O1—Fe—N4—C10	7.8 (2)	C20—N2—C14—N1	−1.0 (3)
N2—Fe—N4—C10	−134.4 (2)	Fe—N2—C14—N1	−165.13 (16)
N3—Fe—N4—C10	−85.1 (2)	C20—N2—C14—C13	178.6 (2)
N5—Fe—N4—C10	−175.1 (3)	Fe—N2—C14—C13	14.5 (3)
O2—Fe—N5—C1	−78.8 (3)	C15—N1—C14—N2	1.7 (3)
O1—Fe—N5—C1	−164.0 (4)	C15—N1—C14—C13	−177.8 (2)
N2—Fe—N5—C1	16.1 (3)	C12—C13—C14—N2	175.8 (3)
N4—Fe—N5—C1	179.7 (3)	N3—C13—C14—N2	−3.6 (3)
N3—Fe—N5—C1	91.7 (3)	C12—C13—C14—N1	−4.7 (4)
O2—Fe—N5—C3	98.43 (18)	N3—C13—C14—N1	176.0 (2)
O1—Fe—N5—C3	13.2 (5)	C14—N1—C15—C16	177.8 (3)
N2—Fe—N5—C3	−166.64 (18)	C14—N1—C15—C20	−1.7 (3)
N4—Fe—N5—C3	−3.04 (17)	N1—C15—C16—C17	179.7 (3)
N3—Fe—N5—C3	−91.02 (18)	C20—C15—C16—C17	−0.9 (4)
C3—N5—C1—S2	0.3 (3)	C15—C16—C17—C18	1.6 (4)
Fe—N5—C1—S2	177.63 (15)	C16—C17—C18—C19	−1.4 (5)
C2—S2—C1—N5	−0.5 (3)	C17—C18—C19—C20	0.6 (4)
C1—S2—C2—C3	0.5 (2)	C14—N2—C20—C19	−178.5 (3)
S2—C2—C3—N5	−0.5 (3)	Fe—N2—C20—C19	−20.2 (4)
S2—C2—C3—C4	178.5 (2)	C14—N2—C20—C15	−0.2 (3)
C1—N5—C3—C2	0.1 (3)	Fe—N2—C20—C15	158.09 (19)
Fe—N5—C3—C2	−177.9 (2)	C18—C19—C20—N2	178.2 (3)
C1—N5—C3—C4	−179.0 (2)	C18—C19—C20—C15	0.1 (4)
Fe—N5—C3—C4	3.0 (3)	C16—C15—C20—N2	−178.4 (2)
C10—N4—C4—N6	−1.2 (3)	N1—C15—C20—N2	1.1 (3)
Fe—N4—C4—N6	−179.71 (17)	C16—C15—C20—C19	0.1 (4)
C10—N4—C4—C3	176.4 (2)	N1—C15—C20—C19	179.6 (2)

### Hydrogen-bond geometry (Å, °)

**Table:**

*D*—H···*A*	*D*—H	H···*A*	*D*···*A*	*D*—H···*A*
O1—H1C···O3^i^	0.75	2.01	2.742 (3)	163
O1—H1B···O4	0.83	1.90	2.688 (3)	157
O1—H1B···S3	0.83	2.80	3.3842 (18)	129
N1—H1···O4^ii^	0.86	1.92	2.764 (3)	165
N1—H1···S3^ii^	0.86	2.73	3.431 (2)	139
N6—H6···O5^iii^	0.86	1.90	2.712 (3)	156
N6—H6···S3^iii^	0.86	2.80	3.656 (2)	173

Symmetry codes: (i) −*x*−1, −*y*+1, −*z*+2; (ii) *x*, *y*+1, *z*; (iii) −*x*, −*y*+1, −*z*+2.
